# *Penta*-B_x_N_y_ sheet: a density functional theory study of two-dimensional material

**DOI:** 10.1038/srep31840

**Published:** 2016-08-19

**Authors:** Jiao Li, Xinyu Fan, Yanpei Wei, Gang Chen

**Affiliations:** 1 Laboratory of Advanced Materials Physics and Nanodevices, School of Physics and Technology, University of Jinan, Jinan, Shandong 250022, P. R. China

## Abstract

By using density functional theory with generalized gradient approximation, we have carried out detailed investigations of two-dimensional B_x_N_y_ nanomaterials in the Cairo pentagonal tiling geometry fully composed of pentagons (*penta*-B_x_N_y_). Only *penta*-BN and BN_2_ planar structures are dynamically stable without imaginary modes in their phonon spectra. Their stabilities have been further evaluated by formation energy analysis, first-principles molecular dynamics simulation, and mechanical stability analysis. *Penta*-BN_2_ is superior to *penta*-BN in structural stability. Its stability analysis against oxidization and functional group adsorption as well as its synthesizing reaction path analysis show possibilities in fabricating *penta*-BN_2_ on experiment. Furthermore, the *penta*-BN_2_ could be transferred from metallic to semiconducting by ionizing or covalently binding an electron per dinitrogen. Also, it has been found to have superior mechanical properties, such as the negative Poisson’s ratio and the comparable stiffness as that of hexagonal *h*-BN sheet. These studies on the stabilities, electronic properties, and mechanical properties suggest *penta*-BN_2_ as an attractive material to call for further studies on both theory and experiment.

Since the discovery of graphene on experiment in 2004^1^, the two-dimensional (2D) nanomaterials have gained explosive researches. Besides the technique of mechanical exfoliation of 2D nanostructures from their parent materials—the corresponding layered bulks, the liquid-phase exfoliation, chemical vapor deposition (CVD), hydrothermal synthesis methods *etc*. have also been applied in experimental studies. At this point many planar nanostructures beyond graphene have been obtained^2^,^3^,^4^,^5^. Many exciting unusual properties originated from the quantum confinement have also been confirmed, showing attractive applications and even more revolutionizing many advanced materials.

The structure-property relationship is an important fundamental research issue in the field of 2D materials. The graphene of the atomic monolayer of carbon atoms arranged in a honeycomb lattice gets massless Dirac fermion characters. The Dirac cones located at K and K′ points in Brillouin zone have been protected by both inversion symmetry and time reversal symmetry. Its linear dispersion relationship of π-band at Dirac point makes charge carriers to be continuously tuned between electrons and holes, whose group velocities are comparable with that of light^6^. However, recent progresses in searching novel 2D carbon allotropes have shown that the hexagonal symmetry is not the necessary conditions for Dirac properties^7^,^8^,^9^,^10^,^11^. Topological arrangement of carbon atoms may hybrid *p*_*x*_ or *p*_*y*_ orbitals with *p*_z_ to perturb the isotropic Dirac cones, which may also bring remarkable properties, such as inherent ferromagnetism, high catalytic activity, potential superconductivity, and metal-semiconductor transition^12^,^13^,^14^,^15^,^16^. Carbon pentagon, hexagon, and heptagon are commonly used as building units of the 2D carbon allotropes. The graphene consists of carbon hexagons, which in fact often contains carbon pentagons and heptagons as structural defects such as the well-known Stone-Wales defect^17^. Wang *et al*. recently studied a novel 2D carbon allotrope with distorted Dirac cone by regularly arranging carbon pentagons, hexagons, and heptagons. Though the carbon pentagons need to be separated from each other by their surrounding hexagons to reduce steric stress according to the isolated pentagon rule (IPR) for fullerenes, considerable effort has been made to stabilize fused-pentagon-based non-IPR fullerenes^12^,^18^, which could be rationalized by the “strain-relief” and “local-aromaticity” principles^18^. Also, some non-IPR fullerenes including the pure pentagon-based C_20_ cage have been achieved experimentally^4^,^18^. Surprisingly, a most recent study performed by Zhang *et al*. confirmed the pure pentagon-based 2D material *penta*-graphene resembling the Cairo pentagonal tiling in geometry, which corresponds to the layer structure of T12-carbon bulk^19^. Inspired by this finding, pentagonal sheet materials of CN_2_^20^, hydrogenated silicene^21^, and B_2_C^22^ have been recently reported. The pentagonal arrangement of atoms induces high energy density in CN_2_, superior flexibility and bipolar magnetic properties in hydrogenated silicene, and tunable band gap in B_2_C. In comparison with graphene, lots of inorganic nanosheets also have hexagonal lattice characters, such as the *h*-BN, SiC, MoS_2_ *etc*., while the non-hexagonal cases are to some sense rare. So, the questions could be raised: could the non-hexagonal lattice be stable for most planar or quasi-planar nanostructures; will the non-hexagonal structures bring new electronic properties in view of the structure-property exploration for advanced nanomaterials? As a contribution to these issues, we have carried out a detailed investigation on the pentagonal *penta*-B_x_N_y_ nanosheets. Their thermodynamic and kinetic stabilities have been carefully evaluated. Considering the fabrication conditions, we have also examined their stabilities against oxidization, functional group adsorption, and charge state. The electronic and mechanical properties of the stable *penta*-B_x_N_y_ nanomaterials have also been discussed.

## Results

**Figure 1:**
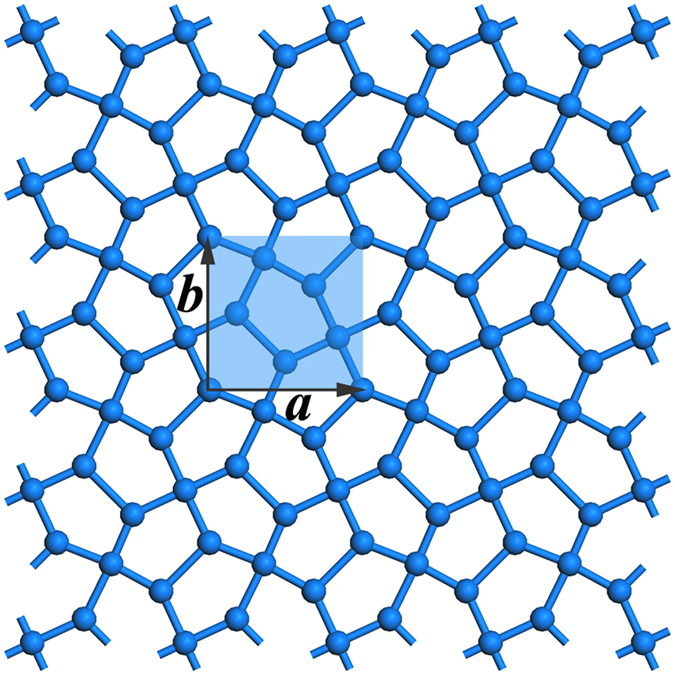
The schematic illustration of Cairo pentagonal tiling geometry with ***a*** × ***b*** repeated unit highlighted. The balls stand for the positions for arranging B or N atoms to form *penta*-B_x_N_y_ nanostructures.

**Figure 2:**
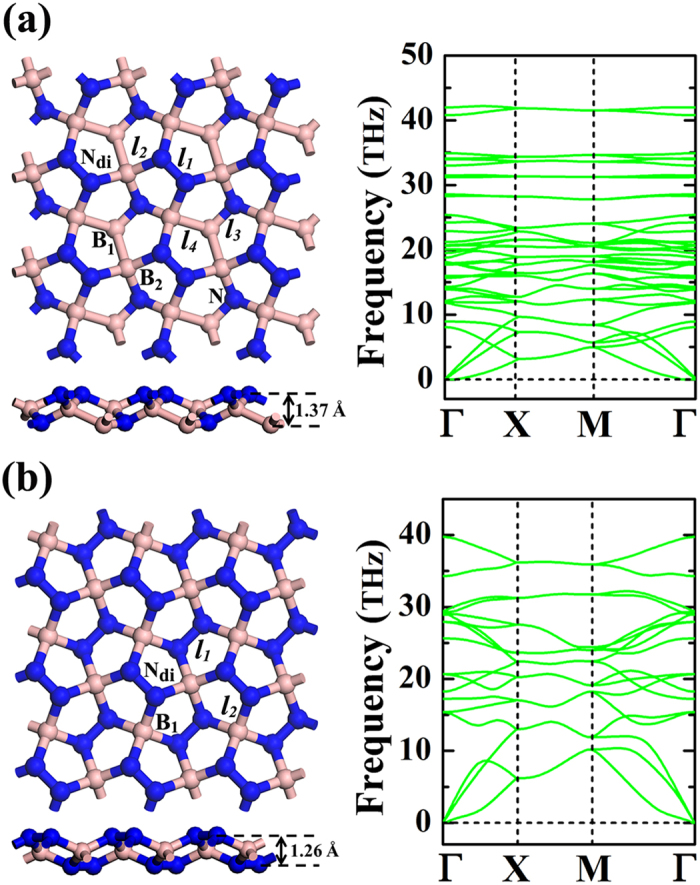
The structures and the calculated phonon spectra for *penta*-BN (**a**) and *penta*-BN_2_ (**b**). The blue and brown balls are for N and B atoms. In (**a**), B_1_ and B_2_ stand for the boron atoms at three- and four-coordinate sites, respectively. N and N_di_ are for the atomic nitrogen atom and dinitrogen. The *l*_*1*_, *l*_*2*_, *l*_*3*_, and *l*_*4*_ are used to stand for the non equivalent bonds, respectively. In (**b**), B_1_ and N_di_ stand for the atomic boron atom and dinitrogen composition species of *penta*-BN_2_, respectively. Accordingly, the *l*_*1*_ and *l*_*2*_ account for the non equivalent bonds.

The Cairo pentagonal tiling is the structural geometry fully composed of pentagons, which is schematically shown in Fig. 1 with the repeated unit highlighted by ***a*** × ***b***. Arranging B and N atoms at possible positions in the repeated unit, we have carefully optimized the geometrical structures of *penta*-B_x_N_y_ nanosheets and calculated their phonon spectra, respectively. In our studies, the number of B atoms ranges from 0 to 6, which simultaneously requires that of N atoms to reversely change from 6 to 0 to meet the requirement of 6 atoms in the primitive unit cell. Only BN (B:N = 3:3) and BN_2_ (B:N = 2:4) sheets are found to be dynamically stable without imaginary modes, which are presented in Fig. 2. Unlike the Cairo pentagonal tiling, the *penta*-BN and BN_2_ sheet structures are found to be slightly buckled to release local steric strain. The *penta*-BN consists of four atomic layers which are the top layer of dinitrogen, the second layer of boron, the third layer of atomic nitrogen, and the bottom layer of boron again, resulting in four different groups of atoms as marked in Fig. 2a. The atomic arrangement shows CM space symmetry and the thickness between top and bottom layers is measured to be 1.37 Å. As tabulated in Table 1, the charge populations on B_1_, B_2_, dinitrogen, and atomic nitrogen species are 2.2, 1.5, 11.9, and 7.0 electrons calculated by Bader analysis^23^,^24^, respectively. The lengths are 1.34, 1.60, 1.36, and 1.78 Å for the *l*_*1*_, *l*_*2*_, *l*_*3*_, and *l*_*4*_ bonds, respectively. However, in the *penta*-BN_2_ sheet, there are only two composition species. The atomic B atoms form one atomic layer being sandwiched between the top and bottom dinitrogen layers, showing 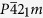 layer group symmetry. The thickness is 1.26 Å. The boron atom and dinitrogen are calculated to have 1.0 and 12.1 electrons. The *l*_*1*_ and *l*_*2*_ bonds are 1.34 and 1.55 Å in length. For the *penta*-BN and BN_2_ nanosheets, the in-plane lattice constants are calculated to be 3.75 and 3.63 Å, respectively.

**Figure 3:**
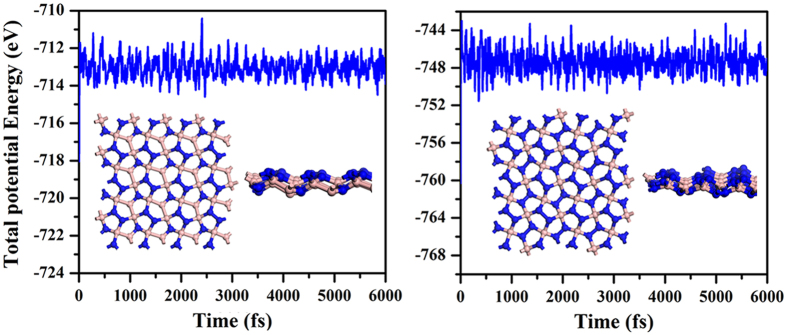
The evolution of potential energy versus the simulation time. The left and right panels are for the molecular dynamics simulations for *penta*-BN at 450 K and *penta*-BN_2_ at 1000 K, respectively. The insets are the geometrical structures obtained at the end of the corresponding simulation studies, respectively.

For the kinetically stable *penta*-BN and BN_2_ sheets, we have also evaluated the melting temperatures to estimate their heat stabilities by using the first-principles molecular dynamics (MD) simulations. In order to minimize the special constraints due to the periodic conditions, the method of supercell has been adopted in our MD simulations to explore the possibilities in structure reconstruction or melting. The energy barrier protecting the geometrical structure to stay at the local minima on potential surface could be estimated by checking whether the structure reconstruction would happen during the MD simulations. The MD simulations have been performed by heating structures to the temperature of 300 K, with an increase of 50 K for the successive simulations. For each study, the simulation lasts for 6 ps with time step of 1 fs. At the end of each simulation, the final structure has been carefully examined. As shown in Fig. 3, the *penta*-BN could withstand the temperature as high as 450 K, while the melting of *penta*-BN_2_ would not occur below the temperature of 1000 K.

**Figure 4:**
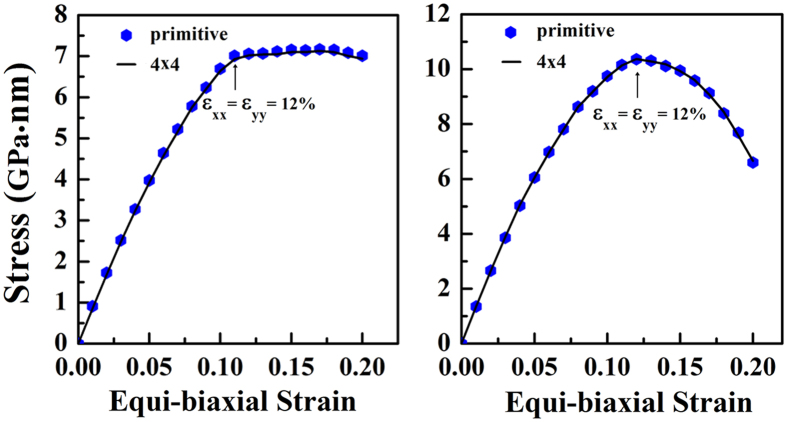
The calculated stress versus the applied equi-biaxial strain for *penta*-BN (left) and BN_2_ (right). The hexagons and solid lines are for the data calculated by using primitive unit cell and 4 × 4 supercell, respectively.

**Figure 5:**
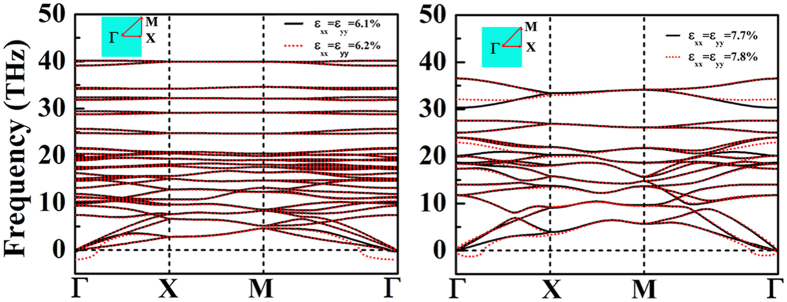
The calculated phonon spectra for *penta*-BN and BN_2_ nanostructures at the corresponding extremes of equi-biaxial strains, respectively.

In Fig. 4, the mechanical effects on the stabilities of *penta*-BN and BN_2_ nanostructures have been estimated. Beside the primitive unit cell, the 4 × 4 supercell has been adopted to release the special constraints due to the periodic conditions. Our calculations clearly show that both of them could withstand biaxial strain up to 12% before the structures start to collapse, suggesting nice static stabilities. However, before mechanical failure, phonon instability known as Kohn anomaly might occur^25^. After applying tensile strain, we have also calculated phonon spectra correspondingly. As shown in Fig. 5, one of the acoustical phonon branches start to have imaginary modes for *penta*-BN under 6.2% strain and *penta*-BN_2_ under 7.8% strain, whose deformation energies are calculated to be 0.58 eV and 1.06 eV, respectively. These suggest *penta*-BN_2_ to be stiffer than *penta*-BN, agreeing with the conclusions obtained in our MD simulations. The melting temperature for *penta*-BN is quite lower as compared with that of *penta*-BN_2_. Also, by applying the finite distortion method^19^, we have calculated the linear elastic constants. Following the standard Voigt notation^26^, the elastic strain energy per unit area can be written as


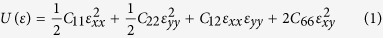


where 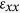 and 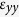 are the uniaxial strains respectively applied along *x* and *y* directions, and 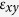 is the equi-biaxial strain. *C*_11_, *C*_22_, *C*_12_, and *C*_66_ are the components of elastic modulus tensor, which could be obtained by calculating the second partial derivative of strain energy with respect to strain. According to Born-Huang criteria^27^, the mechanically stable 2D nanostructure requires the elastic constants to satisfy 
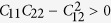
 and 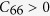. Our calculated elastic constants are presented in Table 2. One can see that both *penta*-BN and BN_2_ are mechanically stable. By calculating the 
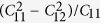
, we have also estimated the in-plane Young’s moduli in Table 2. The *penta*-BN_2_ has a value of 224 N/m being close to the 271 N/m of *h*-BN monolayer, which is stiffer than the *penta*-BN, being in the line of the above studies^28^. Interesting, the Poisson’s ratio C_12_/C_11_ = −0.03 of *penta*-BN_2_ is negative to render it attractive in view of both scientific and technological investigations^29^.

## Discussion

**Figure 6:**
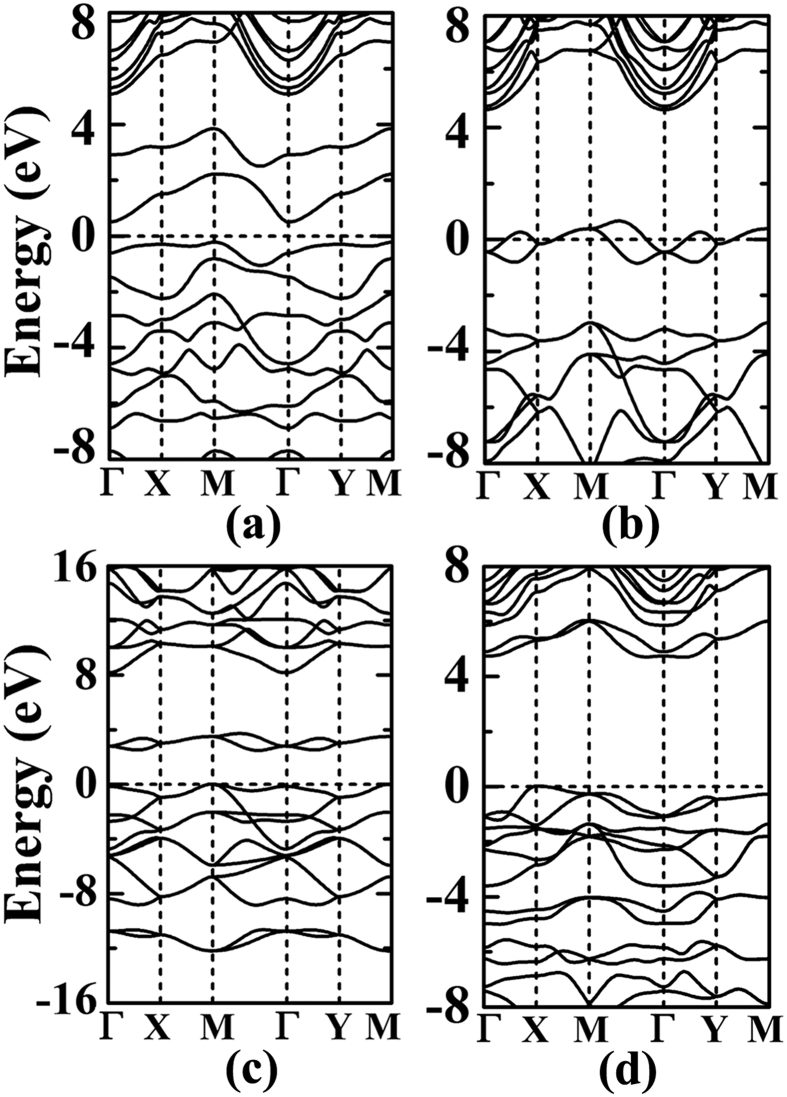
The bandstructures for *penta*-BN (**a**) and BN_2_ (**b**). The (**c**,**d**) are for *penta*-BN_2_ with one electron per dinitrogen to be ionized and the one with full coverage of hydroxyl radical adsorption.

The *penta*-BN consists of the fragments of 3-coordinate B atom, 4-coordinate B atom, dinitrogen N_di_, and atomic N, which have 6.85, 4.60, −0.70, and 7.86 eV formation energies as calculated by below formula.


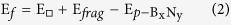


where E_*p*-BxNy_, E_*frag*_, and E_□_ are the total energies calculated for the *penta*-B_x_N_y_ nanostructure, the composition fragment, and the vacancy defected *penta*-B_x_N_y_ structure optimized after removing the fragment. In the calculation, a supercell of 5 × 5 has been adopted to minimize the structural deformation effects from the neighboring images of vacancy defect. Though the reactions of the atomic B and N atoms are exothermic, the reaction of dinitrogen is endothermic as referred to N_2_ molecule. As to its electronic properties, our bandstructure calculations show *penta*-BN to be indirect band gap semiconductor (see Fig. 6a). Its conduction band minimum and valence band maximum are located at Γ and M points, respectively. Here, we must point out that the composition fragments include both dinitrogen and atomic nitrogen, which in combination with the above studied stabilities may challenge its fabrication on experiment.

In comparison, the *penta*-BN_2_ nanostructure is composed of only atomic boron and dinitrogen fragments, which is in form similar to the experimentally fabricated Ti_8_C_12_ metallo-carbohedrene composed of only Ti atoms and C_2_ dimers^30^. Similarly, our previous studies of calcium metal cabides also support the C_2_ dimers as preferable composition fragments^31^,^32^. The composition of dinitrogen could also be seen in the recently reported carbon nitride materials^33^,^34^. These studies shed light on the possibilities in synthesizing the dinitrogen composed *penta*-BN_2_ nanostructure. The formation energies of B atom and dinitrogen compositions of *penta*-BN_2_ are calculated to be 10.77 and 0.04 eV, respectively, showing exothermic reaction properties. Besides, we have vertically displaced a single B atom or dinitrogen from the planar structure step by step. At each step, by freezing the vertical distance between the displaced fragment and the nanosheet, the total energy has been calculated after structural optimization. The energy barriers are found to be 4.40 and 2.04 eV to remove a single B atom and dinitrogen, respectively, supporting the structural stability of *penta*-BN_2_. Also, we have estimated the possibility in synthesizing it by the reaction channel through introducing atomic boron species into the source of nitrogen molecules such as the liquid nitrogen. Actually, a similar fabrication method was previously applied to successfully synthesize transition metal nitrides by compressing metal and N_2_ molecules at high pressure^35^,^36^. A boron atom could bind three N_2_ molecules in maximum through exothermic reaction to form B(N_2_)_3_ complex, which could then meet each other by overcoming 0.68 eV energy barrier to produce the pentagonal building block of *penta*-BN_2_ nanostructure, indicating the possibility for its experimental fabrication.

The bandstructure of *penta*-BN_2_ presented in Fig. 6b shows conducting properties. Its work function is calculated to be 3.3 eV being comparable to those of simple metals such as the 3.68 eV of Mg and the 4.28 eV of Al. When supporting on substrate, the *penta*-BN_2_ might donate electrons, which would in turn affect its electronic properties. After ionizing one electron per dinitrogen, we have again calculated the electronic properties, which suggest the semiconducting properties as shown in Fig. 6c. Due to the fact that the dinitrogens are located on the outer sides to be naked in the quasi-planar nanostructure, they may be capped by functional groups in fabrication, which may affect the structural stability. In our studies, the hydroxyl group is adopted to check the adsorption effects, which may gain presence in experimental studies^37^,^38^. By adsorbing hydroxyl groups on the *penta*-BN_2_ nanostructure, we have carried full optimization of geometrical structure. Then, the optimized structure has been forwarded to carry out MD simulations which suggest the thermal stability up to 1400 K (>1000 K of the melting temperature of free-standing *penta*-BN_2_), hinting enhanced stability. Also, for the optimized structure fully covered by hydroxyl groups, we have calculated its bandstructure and shown in Fig. 6d. For the adsorption configuration, the Bader charge analysis does not show obvious charge donation from dinitrogen to hydroxyl group. The interatomic distance is 1.45 Å between the O and its nearest N, which is only 6% longer than the sum of atomic radii of O and N atoms. In our charge density study, the charge accumulation could be clearly seen between hydroxyl radical and dinitrogen. Furthermore, we have estimated the localization of electrons of the hydroxyl group adsorbed *penta*-BN_2_ by using the electron localization function (ELF) analysis^39^,^40^,^41^, which was introduced in quantum chemistry to measure the parallel spin correlation by defining conditional probability of finding an electron in the neighborhood of another electron with the same spin. ELF is defined as


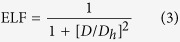



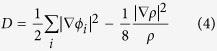



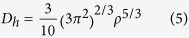


where 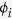 is the Kohn-Sham orbital, and *ρ* is the local density. The ELF data of 0.7 between the dinitrogen and the adsorbed hydroxyl radical supports weak localization of electrons along H-O bond. Therefore, the bonding between dinitrogen and hydroxyl radical should be covalent-like. One of the electrons of the highest occupied molecular orbital of the dinitrogen would be bound in the covalent bond, making the conducting property transition from conducting to semiconducting as shown in Fig. 6.

**Figure 7:**
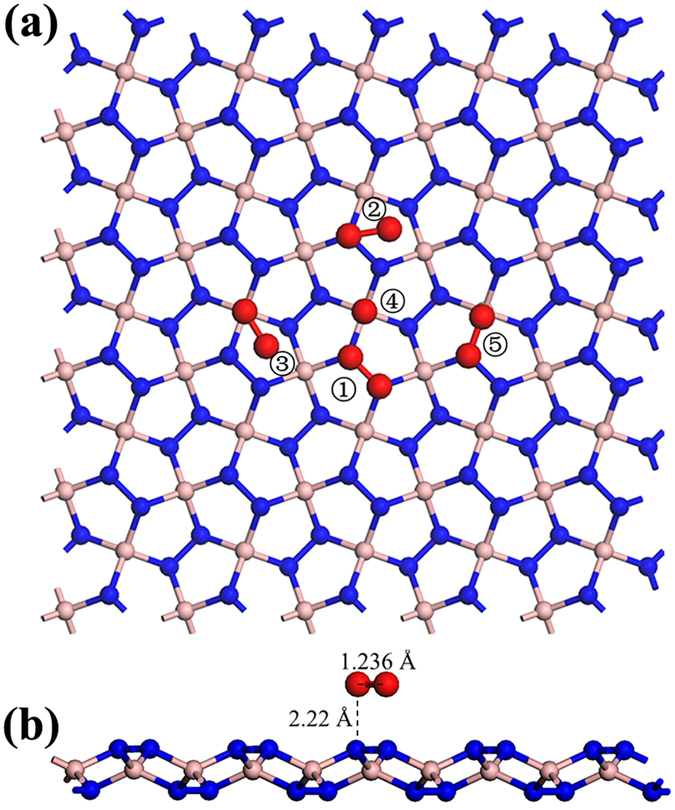
The schematic illustration of the studied O_2_ adsorption configurations (**a**). In (**b**), the side view the lowest energy adsorption configuration is presented. The blue, brown, and red balls are for N, B, and O atoms, respectively.

Besides the effects of charge states and functional group adsorption, the oxidization of *penta*-BN_2_ nanostructure also needs to be discussed considering the fabrication and application in the presence of oxygen. A supercell of 5 × 5 has been employed for studying the adsorption and dissociation of a single O_2_ molecule. The adsorption configurations as shown in Fig. 7 are fully relaxed. The oxygen molecule could parallel cap upon N-N dinitrogen. The O-O bond length would be slightly elongated by 0.5% and the length of the underlying N-N bond would be reduced by 0.7%. The charge gain of O_2_ is 0.1 electrons and the charge depletion of the underlying dinitrogen is also 0.1 electrons. Actually, in our studies, we have also calculated the adsorption of H_2_, N_2_, F_2_, NO, and CO gas molecues. The slight charge transfer only happens in the O_2_ adsorption. This may be attributed to the fact that only O_2_ molecule has the lowest unoccupied molecular orbital to be slightly lower in binding energy than the valence band maximum of *penta*-BN_2_. In the process of band alignment, nearly neglected charge is transferred from dinitrogen to O_2_ molecule. However, the interatomic distance between O and its nearest N is 2.22 Å being of 63% larger than their atomic radius sum^42^, excluding the possibility of obvious orbital overlap between them. Besides the weak overlap indicated by the weak charge transfer, the calculated binding energy of ~0.2 eV for O_2_ adsorption may also include the Coulomb energy of the charged dioxygen felt in the local electric field surrounding dinitrogen. The charge accumulation on dinitrogen as presented in Table 1 would induce a local electric field to affect the adsorption of charged or polarized molecules, being like the case of H_2_ adsorption on C_60_(OM)_12_ (M = Li and Na) clusters^38^. We have used the climbing image nudged elastic band method to study O_2_ dissociation^43^,^44^,^45^,^46^, whose activation energy is calculated to be >2.6 eV to hinder the oxidization of *penta*-BN_2_. In our studies, we have also investigated the structural stability in the conditions of oxygen molecules. After putting one O_2_ molecule upon each dinitrogen, the first-principles molecular dynamics simulation has been carried out at 300 K by using the 4 × 4 supercell. The O_2_ would however start to leave the *penta*-BN_2_ at ~1 ps in our MD simulation. Based on these studies, we would like to conclude that the oxidization of *penta*-BN_2_ sheet structure is not easy.

In summary, the new two-dimensional nanostructures of B_x_N_y_ with Cairo pentagonal tiling geometry have been carefully investigated. Only *penta*-BN and BN_2_ are found to be kinetically stable which do not have imaginary modes in the calculated phonon spectra. Besides, we have also discussed their stabilities from the sides of: (1) the formation energies of structural composition fragments; (2) the thermal stabilities indicated by the melting temperatures found in our molecular dynamics simulations; and (3) the mechanical stabilities to sustain mechanical strain. The *penta*-BN_2_ composed of only atomic boron and dinitrogen species has superior stability than *penta*-BN, which may be synthesized by introducing atomic boron atoms into the source of nitrogen molecules such as the liquid nitrogen. Also, our studies on the oxygen molecule adsorption and its dissociation suggest the stability of *penta*-BN_2_ sheet against oxidization. Its stability has also been evaluated against hydroxyl group adsorption. The bandstructure study of *penta*-BN_2_ sheet shows conducting properties. Due to the charge accumulation, the dinitrogen may tend to donate electrons when the sheet structure of *penta*-BN_2_ is supported on substrate. By ionizing one electron per dinitrogen, the charged *penta*-BN_2_ would be changed to be semiconducting. For the hydroxyl group adsorbed *penta*-BN_2_, each hydroxyl group could bind one electron of each dinitrogen in the covalent-like bond, which could also make the metal-semiconductor transition. Here, we would like also to mention Yagmurcukardes *et al*.’s studies of pentagonal B_2_N_4_ and B_4_N_2_ on the bandstructures and mechanical properties (for example, the stiffness)^47^. In comparison, we have investigated all the possible pentagonal monolayer structure candidates by changing composition species in the primitive unit cell. Only *penta*-BN and BN_2_ are found to be dynamically stable. As for the *penta*-BN_2_ which was also previously investigated by Yagmurcukardes and coworkers^47^, more detailed studies on its structural stability have been performed, for example, the thermal stability estimated by first-principles molecular dynamics simulations, the thermodynamic stability evaluated by formation energy analysis. Besides, considering the potential usages, we have also carefully studied its stability against oxidization and functional group adsorption. The effects of substrate and functional group adsorption on its transport properties are also discussed in our studies. Furthermore, in order to facilitate experimental fabrication, we have also carried out synthesizing reaction path analysis. So, we would like to conclude that our studies contribute to give more comprehensive theoretical results on the possible pentagonal boron nitride monolayer materials, including detailed stability analyses, effects of oxidization and functional group adsorption, transport property modifications, and potential synthesizing reaction path investigations.

## Methods

Our first-principles calculations were performed within the framework of density functional theory with a plane wave basis set as implemented in the Vienna *ab initio* simulation package (VASP)^48^,^49^. The cutoff energy for plane-wave basis set was chosen to be 400 eV. The projector augmented-wave (PAW) method was used^49^. The exchange and correlation energy was described by the generalized gradient approximation with Perdew, Burke, and Ernzerhof (PBE) parameterization^50^. The nanostructure of *penta*-B_x_N_y_ was placed in *xy* plane of the supercell with a 15 Å vacuum in *z* direction, which is large enough to ignore the effects from its neighboring images. The Monkhorst-Pack *k*-mesh of 15 × 15 × 1 was applied to sample *k* points in the first Brillouin zone for integrating electronic properties^51^. All the atoms were fully relaxed with force converge up to 0.02 eV/Å. The calculated total energy was converged to 10^−5^ eV. The first-principles molecular dynamics (MD) simulations lasted for 6 ps with time step of 1 fs. The MD simulations were preformed starting from the temperature of 300 K. Due to the intensive computing loading of MD simulations, the melting temperature was only estimated with the precision of 50 K. Our Bader charge analysis was carried out by using the code developed by Henkelman *et al*.^24^,^52^,^53^. Phonon properties were calculated with the finite displacement method as implemented in Phonopy^54^. In calculating the phonon spectra, the energy convergence criteria were set to 10^−8^ eV for total energy and 0.1 meV/Å for Hellmann-Feynman force.

## Additional Information

**How to cite this article**: Li, J. *et al*. *Penta*-B_x_N_y_ sheet: a density functional theory study of two-dimensional material. *Sci. Rep*. **6**, 31840; doi: 10.1038/srep31840 (2016).

## Figures and Tables

**Table 1:** The calculated lattice constants (Å), bond lengths (Å), and Bader charge populations (e) for the *penta*-BN and BN_2_ nanostructures.

Structure	Lattice	Bond Length				Charge			
		*l*_*1*_	*l*_*2*_	*l*_*3*_	*l*_*4*_	B_1_	B_2_	N_di_	N
BN	a = b = 3.75	1.34	1.60	1.36	1.78	2.16	1.45	11.88	7.04
BN_2_	a = b = 3.63	1.34	1.55	—	—	0.95	—	12.1	—

The studied bonds and the composition species considered in Bader analysis are illustrated in Fig. 2.

**Table 2:** The elastic constants *C*_11_, *C*_22_, *C*_12_, and *C*_22_ in the unit of N/m and the calculated Poisson’s ratio *ν*.

Structure	*C*_11_	*C*_22_	*C*_12_	*C*_66_	*ν*
BN	133.67	133.67	5.48	65.60	0.04
BN_2_	224.18	224.18	−7.03	120.48	−0.03
